# Predicting patient-reported outcomes of radiofrequency uvulopalatoplasty with tonsillectomy in adult obstructive sleep apnea

**DOI:** 10.1007/s11325-025-03366-4

**Published:** 2025-05-22

**Authors:** Samuel Tschopp, Danilo Esaltato, Kurt Tschopp, Khalid Azalmad, Marco Caversaccio, Urs Borner

**Affiliations:** 1 Department of Otorhinolaryngology, Head and Neck Surgery, Inselspital, University Hospital and University of Bern, Freiburgstrasse 20, Bern, 3010 Switzerland; 2 Department of Otorhinolaryngology, Head and Neck Surgery, Kantonsspital Baselland, Liestal, Switzerland; 3 Faculty of Biology and Medicine, University of Lausanne, Lausanne, Switzerland

**Keywords:** Obstructive sleep apnea, Uvulopalatopharyngoplasty, Tonsillectomy, Upper airway surgery, Patient-reported outcome measure, PROM

## Abstract

**Purpose:**

Uvulopalatopharyngoplasty with tonsillectomy is one of the most commonly performed procedures for sleep-disordered breathing, with most studies focusing on objective sleep measurement outcomes. Daytime sleepiness and snoring are important patient-reported outcome measures (PROMs); however, little is known about patient-specific predictors to individualize treatment and guide patient counseling.

**Methods:**

Patients undergoing radiofrequency uvulopalatoplasty with tonsillectomy between 2015 and 2021 were retrospectively analyzed. Patients underwent a standardized clinical head and neck examination. Preoperatively and three months after surgery, sleep apnea testing and questionnaires were administered. Daytime sleepiness and snoring were evaluated using the Epworth Sleepiness Scale (ESS) and a visual analog scale (VAS, 0–10) preoperatively and three months postoperatively. Primary endpoints were predictors influencing ESS and snoring reduction.

**Results:**

In total, 142 patients with a mean age of 47 ± 12 years have been analyzed. ESS significantly decreased from 8.4 ± 4.7 to 4.1 ± 3.0 (*p <* 0.01), and snoring VAS from 7.9 ± 2.0 to 3.3 ± 2.3 (*p <* 0.01). Higher preoperative ESS and snoring scores significantly predicted greater postoperative improvements. Anatomical parameters and indices from sleep studies did not consistently predict outcomes. A greater reduction in the apnea-hypopnea index was associated with ESS reduction but not with snoring intensity improvements.

**Conclusion:**

Radiofrequency uvulopalatoplasty with tonsillectomy significantly reduces daytime sleepiness and snoring severity in adult patients with sleep-disordered breathing. Baseline symptom severity was the sole consistent predictor for PROM improvements, highlighting the multifactorial nature of subjective outcomes and underscoring the necessity for individualized patient counseling and expectation management.

**Supplementary Information:**

The online version contains supplementary material available at 10.1007/s11325-025-03366-4.

## Introduction

Uvulopalatopharyngoplasty with tonsillectomy (UPPP) is the most commonly performed surgical intervention for obstructive sleep apnea and snoring [1, 2]. Traditionally, surgical outcomes have been evaluated primarily using objective measures, notably the apnea-hypopnea index [2]. However, many patients prioritize subjective symptom relief. For individuals with snoring or mild obstructive sleep apnea, improvements in snoring intensity and daytime sleepiness often influence their decision to undergo UPPP. Snoring, in particular, can lead to significant social consequences and negatively affect the sleep quality of bed partners [3, 4]. Additionally, excessive daytime sleepiness markedly impairs daily functioning and impacts quality of life [5–7]. Consequently, evaluating these patient-reported outcomes is essential and of great clinical importance.

Most research predominantly addresses changes in the apnea-hypopnea index. However, objective sleep testing might not adequately capture patient symptoms [6, 8]. Patient-reported outcome measures (PROMs), such as daytime sleepiness [6] and snoring severity [8], have demonstrated weak correlations with objective sleep testing and remain under-investigated following UPPP [9]. A large randomized controlled trial by Browaldh et al. showed significant improvements in daytime sleepiness and quality of life following UPPP with tonsillectomy [5]. Additionally, several studies evaluating various modifications of UPPP have reported similar enhancements in snoring and daytime sleepiness [10–16]. Nonetheless, predictors for individual patient success remain unclear.

To date, little is known about patient-specific predictors, which are crucial to inform personalized patient counseling regarding anticipated improvements in PROMs.

This study aims to identify outcome predictors specifically for daytime sleepiness and snoring, emphasizing PROMs since objective sleep measures have already been extensively studied [12, 13].

## Materials and methods

### Patient selection

This study included consecutive adult patients undergoing radiofrequency uvulopalatoplasty with tonsillectomy (rfUPP + TE) between 2015 and 2021 for snoring and obstructive sleep apnea at our institution to evaluate PROMs. Exclusion criteria were combined surgical procedures (except nasal surgery), central sleep apnea, neurological comorbidities, non-consent for data use, and missing primary endpoint data.

The study received approval from the local ethics committee (Ethikkommission Nordwestschweiz EKNZ 2021–02324) and was conducted in accordance with the Declaration of Helsinki [17]. Reporting follows the Strengthening the Reporting of Observational Studies in Epidemiology (STROBE) guidelines [18].

### Data collection

All patients underwent a comprehensive preoperative head and neck examination using standardized documentation. The examination included anthropometric measurements, such as weight and height, and detailed assessments of upper airway anatomy, explicitly focusing on Friedman tongue position [19], pharyngeal webbing, uvula size, Brodsky tonsil grading [20], and nasal septum deviation. In cases of asymmetrical tonsil sizes, the higher-grade tonsil was recorded. Patient-reported symptoms were assessed through standardized questionnaires using the Epworth Sleepiness Scale (ESS) for daytime sleepiness [21] and a visual analog scale (0–10) for snoring intensity. Home sleep apnea tests using respiratory polygraphy (Nox T3, Nox Medical, Reykjavik, Iceland) and peripheral arterial tonometry (WatchPAT^®^, Itamar Medical, Caesarea, Israel) were performed preoperatively and repeated three months postoperatively to evaluate surgical outcomes.

### Surgical procedure

All patients underwent pharyngeal surgery under general anesthesia. An extracapsular cold-steel tonsillectomy was performed in all patients with tonsillar tissue, irrespective of tonsil size. Elongated uvulae were shortened, preferentially utilizing submucosal resection technique whenever feasible. Hemostasis was achieved through bipolar coagulation. The palatopharyngeal arches were subsequently incised to facilitate mobilization and tension release of the soft palate. Finally, radiofrequency ablation (Erbe VIO 3 electrosurgical unit, Tübingen, Germany) was then performed using a bipolar probe, delivering four to five treatments per side of the palate.

### Statistical analysis

Statistical analyses were performed using R version 4.4.3 (R Foundation for Statistical Computing, Vienna, Austria) following guidelines by Steyerberg et al. [22] and the TRIPOD recommendations [23] for model building.

The primary endpoints were the reduction in daytime sleepiness on the ESS and snoring on a VAS, which was measured as the absolute difference between pre- and postoperative values. Secondary endpoints were the responder status to daytime sleepiness and snoring, which were defined analogously to the commonly used Sher criteria for apnea-hypopnea response [24]. For snoring, we considered a patient a responder if postoperative snoring VAS ≤ 3 and reduced by 50% from baseline. ESS responders were defined as a reduction of 50% from baseline and a postoperative ESS < 11.

Univariate analysis was performed with linear regression models for the ESS and snoring reduction for each biologically plausible variable. Univariate logistic regression models assessed predictors for achieving responder status. P values were adjusted for multiple testing using the Benjamini-Hochberg method [25] and considered significant below 0.05. For the multivariate analyses, missing data were handled through multiple imputations, employing predictive mean matching for numerical variables, logistic regression for binary variables, and polytomous regression for categorical variables over 50 iterations [26]. Predictors were selected using backward stepwise regression, which was applied to the stacked imputed datasets and pooled according to Rubin’s rules. The multivariate logistic regression model underwent bootstrapping for internal validation to adjust for optimism and provide corrected performance estimates.

## Results

### Patient characteristics

Of 306 patients undergoing rfUPP + TE, 142 had a complete data set and were included in the analysis. The median follow-up was 102 days (interquartile range 91–136 days). Table 1 gives an overview of the baseline characteristics. Online Resource Table S1 compares patients included and lost to follow-up, showing no significant differences. Complete data for the snoring follow-up were available in 109 patients, and for ESS in 138 patients. Detailed pre- to postoperative parameters are given in Table 2, showing a significant overall reduction in objective and subjective parameters. A subgroup analysis of patients with (*n* = 62) and without (*n* = 80) concomitant nasal surgery was performed (Online Resource Table S2). Besides impaired nasal breathing and septal deviation, pharyngeal webbing was the only significant difference between the patients with and without nasal surgery. No differences in pre- and postoperative sleep testing and PROMs were observed.

**Table 1:** Baseline characteristics of the study population. Continuous variables are presented as mean ± standard deviation, and categorical variables are given as numbers (percentages)

Number of patients	142
Age (years)	47.1 ± 11.8
Gender, female	13 (9%)
Height (cm)	177.0 ± 8.2
Weight (kg)	89.2 ± 14.1
Body mass index (kg/m2)	28.5 ± 4.2
Neck circumference (cm)	41.3 ± 3.5
Impaired nasal breathing	33 (48%)
Tonsil grade	
0	0 (0%)
1	39 (28%)
2	72 (51%)
3	27 (19%)
4	4 (3%)
Friedman tongue position	
1	14 (11%)
2	43 (36%)
3	45 (35%)
4	26 (20%)
Friedman stage	
1	9 (8%)
2	60 (56%)
3	38 (36%)
4	0 (0%)
Pharyngeal webbing	
normal	51 (38%)
mild	55 (40%)
moderate	30 (22%)
Uvula	
normal	37 (27%)
long	55 (40%)
wide	12 (9%)
long and wide	33 (24%)
Tongue base hyperplasia	
normal	45 (76%)
mild	12 (20%)
moderate	2 (3%)
Epiglottis form	
normal	49 (69%)
omega-shaped	12 (17%)
retroflected	10 (14%)
Occlusion Angle	
Class 1	13 (21%)
Class 2 A	1 (2%)
Class 2B	49 (78%)
Class 3	0 (0%)
Nasal septum deviation	50 (63%)
Turbinate hypertrophy	31 (51%)

**Table 2:** Pre- and postoperative sleep study results and Patient-Reported outcome measures. Data are presented as mean ± standard deviation. Comparisons between pre- and postoperative values were performed using paired t-tests, with P values adjusted for multiple comparisons using the Benjamini-Hochberg method

	Preoperative	Postoperative	Absolute Difference	Relative Difference (%)	*p* value adjusted
Epworth Sleepiness Scale (0–24)	8.4 ± 4.7	4.1 ± 3.0	-4.4	-52	< 0.01
Snoring Index (VAS 0–10)	7.9 ± 2.0	3.3 ± 2.3	-4.6	-58	0.02
Body mass index (kg/m2)	28.6 ± 4.3	28.5 ± 4.1	0	0	< 0.01
Recording time (hours)	7.2 ± 1.4	7.4 ± 1.1	0.3	4	0.08
Apnea-hypopnea index (events/hour)	23.8 ± 18.5	16.4 ± 14.9	-7.4	-31	< 0.01
Apnea index (events/hour)	9.2 ± 12.9	4.2 ± 7.6	-4.9	-54	< 0.01
Central Apnea-hypopnea index (events/hour)	2.5 ± 4.4	0.9 ± 1.6	-1.6	-63	0.01
Oxygen desaturation index (events/hour)	18.3 ± 18.2	13.2 ± 12.5	-5.1	-28	< 0.01
Mean oxygen saturation (%)	93.3 ± 1.9	93.3 ± 1.9	0	0	0.88
Time below 90% oxygen saturation (%)	6.1 ± 12.5	6 ± 13.2	-0.1	-2	0.88
Cartwright Index	1.8 ± 1.5	1.8 ± 1.3	0	2	0.88
Supine time (% of total sleep time)	38.4 ± 24.9	39.8 ± 25.4	1.4	4	0.31
Apnea-hypopnea index supine (events/hour)	34.5 ± 24.8	26.2 ± 22.7	-8.4	-24	< 0.01
Oxygen desaturation index supine (events/hour)	28.1 ± 24.2	21.5 ± 19.0	-6.6	-24	< 0.01
Heart rate (beats/minute)	62.9 ± 7.4	64 ± 7.6	1	2	0.12

VAS, visual analog scale

## Epworth sleepiness scale

**Fig. 1:**
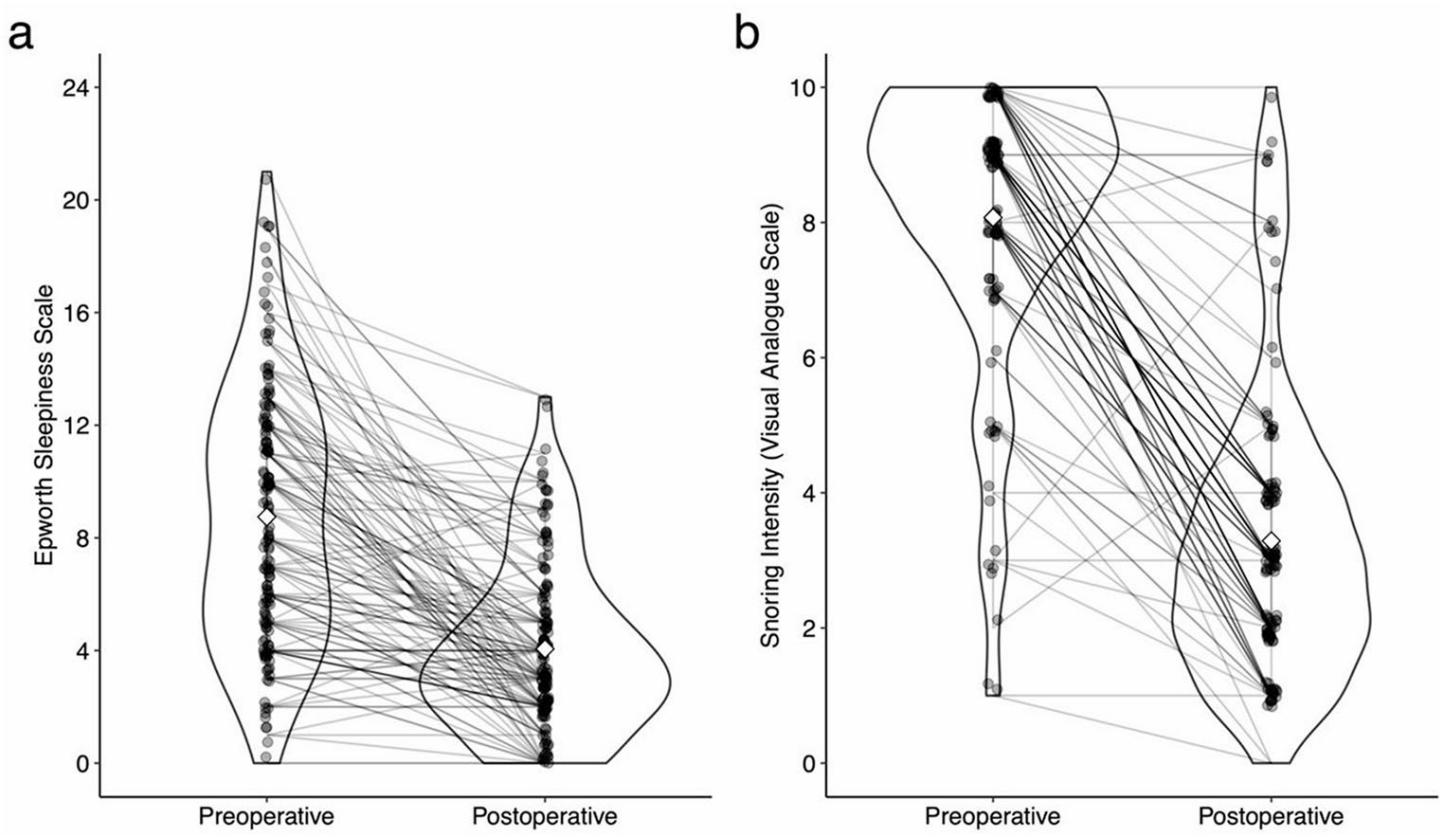
Violin plots illustrating Pre- and Postoperative Values for the Epworth Sleepiness Scale (**a**) and Snoring Intensity on a Visual Analog Scale (**b**). Individual patient data points are represented by dots and connected by lines to show paired changes. Diamonds indicate the mean values of the whole cohort for pre- and postoperative measurements

Across the entire cohort, we observed a significant postoperative improvement in daytime sleepiness, with mean ESS scores decreasing from 8.4 ± 4.7 to 4.1 ± 3.0 (*p* < 0.01; Fig. 1a). Univariate linear regression analysis revealed that a higher preoperative ESS score was significantly associated with greater ESS reduction (β = 0.79; 95% CI: 0.68–0.90; *p* < 0.01; Online Resource Fig. S1). Uncorrected models initially indicated associations between ESS reduction and gender (Online Resource Fig. S2), snoring intensity, preoperative AHI (**Online Resource Fig. S3**), and preoperative apnea index; however, these associations were not significant after correction for multiple comparisons (Table 3). Multivariate analysis demonstrated that adding additional predictors did not enhance the accuracy beyond the univariate model. Notably, the ESS reduction did correlate with the AHI reduction (β = 0.07; 95% CI: 0.02–0.11; *p* = 0.004; **Online Resource Fig. S4**). However, because the reduction in AHI is only known after the surgery, it cannot serve as a predictor.

Among ESS responders, defined as those achieving a postoperative ESS score below 11 with at least a 50% ESS reduction from preoperative, a significant association with the preoperative ESS was found (odds ratio = 1.15; 95% CI: 1.06–1.25; *p* < 0.01, **Online Resource Table S3**). No other predictors or combination of predictors showed significant associations in univariate and multivariate analyses.

**Table 3:** Univariate analysis for predictors of Epworth sleepiness scale reduction. Linear regression models were applied, and P values were adjusted for multiple comparisons using the Benjamini-Hochberg method

Variable	Estimate with 95% CI	*p* value	*p* value adjusted
Age (years)	0.00 (-0.07–0.06)	0.94	0.97
**Gender (female)**	**2.98 (0.34–5.61)**	**0.03**	0.30
Neck circumference (cm)	-0.1 (-0.34–0.14)	0.41	0.87
Height (cm)	-0.04 (-0.15–0.07)	0.43	0.87
Weight (kg)	-0.03 (-0.09–0.03)	0.31	0.83
BMI (kg/m2)	-0.06 (-0.27–0.15)	0.57	0.92
**Epworth Sleepiness Scale preoperative**	**0.79 (0.68–0.90)**	**< 0.01**	**< 0.01**
**Snoring (VAS) preoperative**	**0.45 (0.03–0.87)**	**0.04**	0.30
Impaired nasal breathing	0.56 (-1.81–2.93)	0.64	0.92
Tonsil grade	1.06 (0.05–2.07)	0.04	0.30
Friedman tongue position	0.26 (-0.66–1.18)	0.58	0.92
Friedman stage	-0.8 (-2.38–0.78)	0.32	0.83
Pharyngeal webbing	0.34 (-0.74–1.42)	0.54	0.92
Uvula grade	1.28 (-0.74–3.3)	0.21	0.82
Tongue base hyperplasia	1.44 (-1.21–4.09)	0.28	0.83
Epiglottis form	0.39 (-2.77–3.56)	0.80	0.97
Occlusion angle	3.08 (-7.75–13.91)	0.57	0.92
Septal deviation	-0.12 (-2.37–2.12)	0.91	0.97
Turbinate hypertrophy	-0.13 (-2.59–2.33)	0.92	0.97
*Preoperative Sleep Testing*			
Recording time (hours)	-0.58 (-1.2–0.05)	0.07	0.47
**Apnea-hypopnea index (events/hour)**	**0.04 (0.00–0.08)**	**0.04**	0.30
**Apnea index (events/hour)**	**0.09 (0.02–0.15)**	**0.01**	0.26
Central Apnea-hypopnea index (events/hour)	-0.27 (-0.64–0.09)	0.14	0.59
Oxygen desaturation index (events/hour)	0.01 (-0.05–0.06)	0.83	0.97
Mean oxygen saturation (%)	0.03 (-0.53–0.59)	0.92	0.97
Time below 90% oxygen saturation (%)	-0.02 (-0.09–0.06)	0.68	0.92
Cartwright Index	-0.19 (-0.65–0.27)	0.42	0.87
Supine time (% of total sleep time)	0.01 (-0.02–0.04)	0.37	0.87
Apnea-hypopnea index supine (events/hour)	0.02 (-0.01–0.05)	0.24	0.83
Oxygen desaturation index supine (events/hour)	-0.01 (-0.05–0.03)	0.67	0.92
Heart rate (beats/minute)	0.02 (-0.12–0.15)	0.78	0.97

## Snoring

We observed a significant postoperative reduction in snoring intensity across the entire cohort, decreasing from 7.9 ± 2.0 to 3.3 ± 2.3 (*p <* 0.01; Fig. 1b). Individuals with higher preoperative snoring intensity experienced a more pronounced reduction in snoring (β = 0.73; 95% CI: 0.54–0.92; *p* < 0.01; Table 4, **Online Resource Fig. S5**). Although uncorrected univariate correlations were identified between snoring reduction and both BMI **(Online Resource Fig. S6)** and Friedman Stage **(Online Resource Fig. S7)**, these associations were no longer significant after correcting for multiple comparisons. Notably, the snoring reduction did not correlate with the AHI reduction (β = 0.007; 95% CI: -0.021–0.036; *p* = 0.63; **Online Resource Fig. S8**).

Snoring responders were defined as individuals achieving a postoperative VAS score of ≤ 3 with at least a 50% reduction from baseline. No significant predictors of snoring responders were identified (**Online Resource Table S4**).

**Table 4:** Univariate analysis for the snoring reduction. Linear regression models were applied, and P values were adjusted for multiple comparisons using the Benjamini-Hochberg method

Variable	Estimate with 95% CI	*p* value	*p* value adjusted
Age (years)	-0.02 (-0.06–0.03)	0.51	0.95
Gender (female)	0.84 (-0.97–2.65)	0.36	0.95
Neck circumference (cm)	-0.11 (-0.3–0.08)	0.27	0.95
Height (cm)	0.02 (-0.05–0.08)	0.65	0.95
Weight (kg)	-0.03 (-0.07–0.01)	0.13	0.86
**BMI (kg/m2)**	**-0.14 (-0.27 - -0.01)**	**0.04**	0.56
Epworth Sleepiness Scale preoperative	0.11 (0.00–0.22)	0.05	0.56
**Snoring (VAS) preoperative**	**0.73 (0.54–0.92)**	**< 0.01**	**< 0.01**
Impaired nasal breathing	0.35 (-1.61–2.3)	0.72	0.95
Tonsil grade	0.02 (-1.15–1.19)	0.97	0.98
Friedman tongue position	1.12 (-0.80–3.04)	0.25	0.95
**Friedman stage**	**-1.44 (-2.39 - -0.48)**	**< 0.01**	0.09
Pharyngeal webbing	0.06 (-1.08–1.21)	0.91	0.98
Uvula grade	0.42 (-0.87–1.71)	0.52	0.95
Tongue base hyperplasia	1.12 (-0.80–3.04)	0.25	0.98
Epiglottis form	-0.09 (-2.98–2.79)	0.95	0.98
Occlusion angle	1.38 (-5.59–8.34)	0.69	0.95
Septal deviation	-0.5 (-2.3–1.3)	0.58	0.95
Turbinate hypertrophy	-0.05 (-2.26–2.17)	0.97	0.98
*Preoperative Sleep Testing*			
Recording time (hours)	0.23 (-0.22–0.68)	0.32	0.95
Apnea-hypopnea index (events/hour)	0.01 (-0.01–0.04)	0.37	0.95
Apnea index (events/hour)	0.01 (-0.03–0.06)	0.55	0.95
Central Apnea-hypopnea index (events/hour)	-0.19 (-0.43–0.05)	0.12	0.86
Oxygen desaturation index (events/hour)	0.01 (-0.03–0.05)	0.62	0.95
Mean oxygen saturation (%)	0.07 (-0.34–0.49)	0.72	0.95
Time below 90% oxygen saturation (%)	-0.01 (-0.06–0.04)	0.69	0.95
Cartwright Index	-0.26 (-0.8–0.29)	0.35	0.95
Supine time (% of total sleep time)	0.00 (-0.02–0.02)	0.74	0.95
Apnea-hypopnea index supine (events/hour)	0.01 (-0.02–0.03)	0.55	0.95
Oxygen desaturation index supine (events/hour)	0.01 (-0.02–0.04)	0.59	0.95
Heart rate (beats/minute)	0.03 (-0.06–0.12)	0.54	0.95

## Discussion

Across the entire cohort, we observe a significant improvement in PROMs, with a clinically relevant reduction of daytime sleepiness and snoring intensity following rfUPP + TE in adult patients. The observed improvements in ESS scores and snoring intensity corroborate findings from earlier studies [5, 11, 14]. In our analysis, higher preoperative ESS and snoring intensity scores emerged as the only significant predictors of postoperative reduction. Patients presenting with more pronounced daytime sleepiness or severe snoring preoperatively experienced more substantial symptom relief. Greater preoperative daytime sleepiness increased the odds ratio for being a responder on ESS, whereas snoring responders could not be predicted. Interestingly, no anatomical parameter or sleep-testing index consistently predicted improvements.

The multifactorial nature of subjective symptom relief likely explains this lack of clear anatomical and polysomnographic predictors. Daytime sleepiness and snoring intensity are influenced by numerous factors, including individual sensitivity, perception of sleep quality, lifestyle, psychological status, and even partner-reported sleep disturbances [3, 4, 6, 10]. These variables are typically not captured comprehensively by routine clinical examinations or polysomnography, emphasizing the complexity of accurately predicting surgical outcomes based solely on objective measurements.

Interestingly, objective improvements in AHI were associated with reduced daytime sleepiness, whereas the snoring improvements did not correlate. This might be explained by the finding that nocturnal hypoxemia is a causal factor for daytime sleepiness [27]. Snoring intensity is judged by the beholder’s ear and subject to the psychoemotional disposition of the bedpartner towards the snorer, as shown in the seminal article by Hoffstein et al. [28]. Subjective assessment of snoring has been shown to be highly reliable. However, bed partners might confuse snoring intensity with annoyance caused by nocturnal snoring [29]. For the ESS, the test-retest reliability is relatively low, which might confound the accuracy of the statistical analysis [30]. This further underscores the loose correlation between objective testing and PROMs [6, 8]. Further, night-to-night variability in sleep testing might limit the predictive power and correlation [31, 32].

### Strengths

This study’s strengths include a relatively large and homogeneous patient cohort, comprehensive and systematic preoperative head and neck assessments, and the use of a standardized surgical technique. Importantly, the study is characterized by a structured and consistent postoperative follow-up protocol, incorporating validated and standardized questionnaires to capture PROMs reliably. This systematic approach enhances the robustness and clinical relevance of the findings.

### Limitations

Our study has several limitations. Its retrospective and unblinded design introduces the risk of bias. A considerable proportion of patients were lost to follow-up. While no significant differences were observed between the groups, the results may still be negatively biased, as patients with symptom resolution might be less likely to attend follow-up consultations. The follow-up period was restricted to three months, limiting the ability to assess long-term PROMs. Some patients underwent concomitant nasal surgery, which may be a confounder, despite the sleep testing and PROM outcomes not differing between the groups. The surgical technique using rfUPP + TE may differ from other commonly used techniques, limiting our results’ generalizability. Moreover, the substantial variability inherent in objective sleep studies [31, 32] and subjective PROMs [28–30] may mask subtle predictive associations, warranting further prospective studies with larger cohorts. Due to this retrospective design, no additional PROMs, such as disease-specific quality of life, could be analyzed.

### Future research

Future research should analyze a broader spectrum of PROMs to better depict disease-specific quality of life. Examining additional variables, such as psychometric and environmental factors, could improve patient selection, individualized patient counseling, and enhance surgical outcome predictions. Future research on sleep surgery should routinely incorporate PROMs as endpoints, as they are crucial for patients’ decision-making and essential for patient satisfaction.

## Conclusion

While rfUPP + TE reliably improves daytime sleepiness and snoring, only baseline severity of symptoms predicted the degree of improvement. Anatomical features and sleep testing parameters did not consistently correlate with patient-reported symptoms. Acknowledging the multifactorial basis of daytime sleepiness and snoring is crucial when counseling patients preoperatively and setting realistic expectations.

## Electronic supplementary material

Below is the link to the electronic supplementary material.

Supplementary Material 1

## Data Availability

The data supporting this study’s findings are available upon request from Samuel Tschopp, the corresponding author.
