# Supplementary material for: Predicting patient-reported outcomes of radiofrequency uvulopalatoplasty with tonsillectomy in adult obstructive sleep apnea
Source: Sleep Breath. 2025 May 22;29(3):194. doi: 10.1007/s11325-025-03366-4 (PMC12098473; doi:10.1007/s11325-025-03366-4)
Supplement: Supplementary file 1 — Supplementary Material 1 [file 11325_2025_3366_MOESM1_ESM.docx]

**Predicting Patient-Reported Outcomes of Radiofrequency Uvulopalatoplasty with Tonsillectomy in Adult Obstructive Sleep Apnea**

**Authors:** Samuel Tschopp*, MD^1^; Danilo Esaltato*, MD^1^; Kurt Tschopp, MD^2^; Khalid Azalmad, MD^2,3^; Marco Caversaccio, MD^1^; Urs Borner, MD^1^

**Affiliations:**

1 Department of Otorhinolaryngology, Head and Neck Surgery, Inselspital, University Hospital and University of Bern, Bern, Switzerland

2 Department of Otorhinolaryngology, Head and Neck Surgery, Kantonsspital Baselland, Liestal, Switzerland

3 Faculty of Biology and Medicine, University of Lausanne, Lausanne, Switzerland

**Corresponding author:**

Dr. med. Samuel Tschopp

Department of Otorhinolaryngology, Head and Neck Surgery

Inselspital, University Hospital, and University of Bern

Freiburgstrasse 20

3010 Bern

Switzerland

samuel.tschopp@insel.ch

ORCID: 0000-0002-5666-2092**Table of Content**

**Online Resource Table S1** Comparison of Baseline Characteristics between Included Patients and those Lost to Follow-up.

**Online Resource Table S2** Comparison of Patients with and without Concomitant Nasal Surgery.

**Online Resource Table S3** Univariate Analysis for Predictors of Epworth Sleepiness Scale Responders.

**Online Resource Table S4** Univariate Analysis for Predictors of Snoring Responders.

**Online Resource Fig. S1** Relationship between Epworth Sleepiness Scale reduction and preoperative Epworth Sleepiness Scale scores.

**Online Resource Fig. S2** Association of Epworth Sleepiness Scale reduction with Gender.

**Online Resource Fig. S3** Relationship between Epworth Sleepiness Scale reduction and preoperative Apnea-Hypopnea Index.

**Online Resource Fig. S4** Relationship between Epworth Sleepiness Scale reduction and Apnea-Hypopnea Index Reduction.

**Online Resource Fig. S5** Relationship between Snoring Intensity Reduction and preoperative Snoring Intensity.

**Online Resource Fig. S6** Relationship between Snoring Intensity Reduction and Body Mass Index.

**Online Resource Fig. S7** Association of Snoring Intensity Reduction and Friedman Stage.

**Online Resource Fig. S8** Relationship between Snoring Intensity Reduction and Apnea-Hypopnea Index Reduction.

**Online Resource Table S1** Comparison of Baseline Characteristics between Included Patients and those Lost to Follow-up.

|  | Included | Lost to Follow-up | p value | p value adjusted |
| --- | --- | --- | --- | --- |
| Number of patients | 142 | 164 |  |  |
| Age (years) | 47.1 ± 11.8 | 42.3 ± 10.7 | <0.01 | 0.10 |
| Gender, female | 13 (9%) | 24 (15%) | 0.20 | 0.51 |
| Height (cm) | 177.0 ± 8.2 | 177.2 ± 9.1 | 0.88 | 0.93 |
| Weight (kg) | 89.2 ± 14.1 | 89.9 ± 15.1 | 0.68 | 0.89 |
| Body mass index (kg/m2) | 28.5 ± 4.2 | 28.7 ± 4.5 | 0.72 | 0.89 |
| Neck circumference (cm) | 41.3 ± 3.5 | 41.6 ± 4.0 | 0.51 | 0.88 |
| Epworth Sleepiness Scale | 8.4 ± 4.7 | 8.1 ± 4.9 | 0.23 | 0.51 |
| Snoring index (VAS 0-10) | 7.9 ± 2.0 | 7.8 ± 2.0 | 0.27 | 0.56 |
| Impaired nasal breathing | 33 (48%) | 70 (60%) | 0.13 | 0.51 |
| Tonsil grade |  |  | 0.01 | 0.10 |
| 0 | 0 (0%) | 12 (7%) |  |  |
| 1 | 39 (28%) | 36 (22%) |  |  |
| 2 | 72 (51%) | 81 (49%) |  |  |
| 3 | 27 (19%) | 33 (20%) |  |  |
| 4 | 4 (3%) | 2 (1%) |  |  |
| Friedman tongue position |  |  | 0.20 | 0.51 |
| 1 | 14 (11%) | 12 (8%) |  |  |
| 2 | 43 (36%) | 47 (31%) |  |  |
| 3 | 45 (35%) | 70 (47%) |  |  |
| 4 | 26 (20%) | 21 (14%) |  |  |
| Friedman stage |  |  | 0.02 | 0.16 |
| 1 | 9 (8%) | 15 (11%) |  |  |
| 2 | 60 (56%) | 52 (38%) |  |  |
| 3 | 38 (36%) | 67 (49%) |  |  |
| 4 | 0 (0%) | 3 (2%) |  |  |
| Pharyngeal webbing |  |  | 0.01 | 0.10 |
| normal | 51 (38%) | 32 (21%) |  |  |
| mild | 55 (40%) | 74 (49%) |  |  |
| moderate | 30 (22%) | 45 (30%) |  |  |
| Uvula |  |  | 0.90 | 0.93 |
| normal | 37 (27%) | 46 (30%) |  |  |
| long | 55 (40%) | 60 (40%) |  |  |
| wide | 12 (9%) | 14 (9%) |  |  |
| long and wide | 33 (24%) | 32 (21%) |  |  |
| Tongue base hyperplasia |  |  | 0.17 | 0.51 |
| normal | 45 (76%) | 58 (62%) |  |  |
| mild | 12 (20%) | 31 (33%) |  |  |
| moderate | 2 (3%) | 5 (5%) |  |  |
| Epiglottis form |  |  | 0.78 | 0.93 |
| normal | 49 (69%) | 75 (73%) |  |  |
| omega-shaped | 12 (17%) | 17 (17%) |  |  |
| retroflected | 10 (14%) | 11 (11%) |  |  |
| Occlusion Angle |  |  | 0.72 | 0.89 |
| Class 1 | 13 (21%) | 22 (19%) |  |  |
| Class 2A | 1 (2%) | 3 (3%) |  |  |
| Class 2B | 49 (78%) | 90 (77%) |  |  |
| Class 3 | 0 (0.0%) | 2 (2%) |  |  |
| Nasal septum deviation | 50 (63%) | 90 (74%) | 0.12 | 0.51 |
| Turbinate hypertrophy | 31 (51%) | 56 (55%) | 0.68 | 0.89 |
| *Preoperative Sleep Testing* |  |  |  |  |
| Recording time (hours) | 7.1 ± 1.5 | 7.3 ± 1.3 | 0.31 | 0.57 |
| Apnea-hypopnea index (events/hour) | 25.4 ± 18.8 | 22.4 ± 18.3 | 0.17 | 0.51 |
| Apnea index (events/hour) | 10.4 ± 14.3 | 7.9 ± 11.3 | 0.22 | 0.51 |
| Central Apnea-hypopnea index (events/hour) | 2.4 ± 4.6 | 2.6 ± 4.4 | 0.81 | 0.93 |
| Oxygen desaturation index (events/hour) | 35.5 ± 24.7 | 33.7 ± 24.9 | 0.57 | 0.89 |
| Mean oxygen saturation (%) | 1.8 ± 1.8 | 1.7 ± 1.1 | 0.84 | 0.93 |
| Time below 90% oxygen saturation (%) | 18.3 ± 16.6 | 18.4 ± 19.4 | 0.97 | 0.97 |
| Cartwright Index | 29.0 ± 25.0 | 27.4 ± 23.6 | 0.64 | 0.97 |
| Supine time (% of total sleep time) | 40.4 ± 27.2 | 36.6 ± 22.6 | 0.22 | 0.89 |
| Apnea-hypopnea index supine (events/hour) | 93.1 ± 1.7 | 93.4 ± 2.0 | 0.20 | 0.51 |
| Oxygen desaturation index supine (events/hour) | 7.0 ± 12.9 | 5.3 ± 12.2 | 0.31 | 0.57 |
| Heart rate (beats/minute) | 62.9 ± 7.4 | 62.4 ± 8.6 | 0.60 | 0.89 |

**Online Resource Table S2** Comparison of Patients with and without Concomitant Nasal Surgery.

|  | No nasal surgery | Nasal surgery | p value | p value adjusted |
| --- | --- | --- | --- | --- |
| Number of patients | 80 | 62 |  |  |
| Age (years) | 47.7 ± 12.7 | 46.3 ± 10.6 | 0.48 | 0.83 |
| Gender, female | 7 (9%) | 6 (10%) | 1.00 | 1.00 |
| Height (cm) | 176.1 ± 8.3 | 178.4 ± 7.9 | 0.13 | 0.56 |
| Weight (kg) | 88.5 ± 12.9 | 90.2 ± 15.9 | 0.52 | 0.83 |
| Body mass index (kg/m2) | 28.5 ± 3.7 | 28.4 ± 4.9 | 0.84 | 0.97 |
| Neck circumference (cm) | 41.5 ± 3.5 | 41.1 ± 3.5 | 0.56 | 0.83 |
| *Epworth Sleepiness Scale* |  |  |  |  |
| Preoperative | 8.8 ± 4.1 | 8.8 ± 5.2 | 0.99 | 1.00 |
| Postoperative | 4.3 ± 3.3 | 3.7 ± 2.6 | 0.19 | 0.70 |
| *Snoring (VAS)* |  |  |  |  |
| Preoperative | 8.2 ± 2.0 | 7.8 ± 2.2 | 0.31 | 0.83 |
| Postoperative | 3.6 ± 2.4 | 3.1 ± 2.1 | 0.29 | 0.83 |
| Impaired nasal breathing | 15 (31%) | 18 (86%) | <0.01 | <0.01 |
| Tonsil grade |  |  | 0.07 | 0.50 |
| 0 | 0 (0%) | 0 (0%) |  |  |
| 1 | 17 (21%) | 22 (36%) |  |  |
| 2 | 45 (56%) | 27 (44%) |  |  |
| 3 | 14 (18%) | 13 (21%) |  |  |
| 4 | 4 (5%) | 0 (0%) |  |  |
| Friedman tongue position |  |  | 0.49 | 0.83 |
| 1 | 8 (11%) | 6 (11%) |  |  |
| 2 | 21 (28%) | 22 (41%) |  |  |
| 3 | 28 (38%) | 17 (32%) |  |  |
| 4 | 17 (23%) | 9 (17%) |  |  |
| Friedman stage |  |  | 0.56 | 0.83 |
| 1 | 4 (6%) | 5 (12%) |  |  |
| 2 | 38 (59%) | 22 (52%) |  |  |
| 3 | 23 (35%) | 15 (36%) |  |  |
| 4 | 0 (0%) | 0 (0%) |  |  |
| Pharyngeal webbing |  |  | <0.01 | <0.01 |
| normal | 19 (25%) | 32 (54%) |  |  |
| mild | 39 (51%) | 16 (27%) |  |  |
| moderate | 19 (25%) | 11 (19%) |  |  |
| Uvula |  |  | 0.12 | 0.56 |
| normal | 19 (25%) | 15 (25%) |  |  |
| long | 36 (47%) | 19 (32%) |  |  |
| wide | 7 (9%) | 5 (8%) |  |  |
| long and wide | 15 (20%) | 18 (30%) |  |  |
| Tongue base hyperplasia |  |  | 0.31 | 0.83 |
| normal | 34 (74%) | 11 (85%) |  |  |
| mild | 11 (24%) | 1 (8%) |  |  |
| moderate | 1 (2%) | 1 (8%) |  |  |
| Epiglottis form |  |  | 0.55 | 0.83 |
| normal | 33 (66%) | 16 (76%) |  |  |
| omega-shaped | 10 (20%) | 2 (10%) |  |  |
| retroflected | 7 (14%) | 3 (14%) |  |  |
| Occlusion Angle |  |  | 0.03 | 0.26 |
| Class 1 | 6 (14%) | 7 (37%) |  |  |
| Class 2A | 0 (0%) | 1 (5%) |  |  |
| Class 2B | 38 (86%) | 11 (58%) |  |  |
| Class 3 | 0 (0%) | 0 (0%) |  |  |
| Nasal septum deviation | 26 (50%) | 24 (86%) | <0.01 | <0.01 |
| Turbinate hypertrophy | 18 (41%) | 13 (77%) | 0.03 | 0.26 |
| *Preoperative Sleep Testing* |  |  |  |  |
| Recording time (hours) | 7.4 ± 1.4 | 6.7 ± 1.7 | 0.02 | 0.26 |
| Apnea-hypopnea index (events/hour) | 25.4 ± 19.4 | 25.3 ± 18.1 | 0.99 | 1.00 |
| Apnea index (events/hour) | 11.6 ± 16.4 | 8.9 ± 11.3 | 0.40 | 0.83 |
| Central Apnea-hypopnea index (events/hour) | 1.9 ± 2.8 | 3.5 ± 7.2 | 0.34 | 0.83 |
| Oxygen desaturation index (events/hour) | 18.8 ± 18.1 | 17.4 ± 13.8 | 0.68 | 0.83 |
| Mean oxygen saturation (%) | 93.1 ± 1.9 | 93.2 ± 1.4 | 0.67 | 0.83 |
| Time below 90% oxygen saturation (%) | 7.7 ± 14.0 | 5.8 ± 10.7 | 0.48 | 0.83 |
| Cartwright Index | 1.8 ± 2.2 | 1.8 ± 1.1 | 0.98 | 1.00 |
| Supine time (% of total sleep time) | 40.6 ± 27.7 | 40.1 ± 26.8 | 0.93 | 1.00 |
| Apnea-hypopnea index supine (events/hour) | 34.4 ± 23.8 | 36.8 ± 26.2 | 0.60 | 0.83 |
| Oxygen desaturation index supine (events/hour) | 28.1 ± 25.0 | 30.6 ± 25.3 | 0.65 | 0.83 |
| Heart rate (beats/minute) | 62.7 ± 7.0 | 63.4 ± 8.1 | 0.65 | 0.83 |
| *Postoperative Sleep Testing* |  |  |  |  |
| Recording time (hours) | 7.4 ± 1.0 | 7.5 ± 0.9 | 0.56 | 0.83 |
| Apnea-hypopnea index (events/hour) | 13.9 ± 14.5 | 14.9 ± 12.3 | 0.69 | 0.83 |
| Apnea index (events/hour) | 4.4 ± 9.8 | 3.7 ± 3.8 | 0.67 | 0.83 |
| Central Apnea-hypopnea index (events/hour) | 0.6 ± 1.1 | 1.6 ± 2.5 | 0.02 | 0.26 |
| Oxygen desaturation index (events/hour) | 12.1 ± 13.3 | 13.5 ± 11.4 | 0.58 | 0.83 |
| Mean oxygen saturation (%) | 93.1 ± 2.1 | 92.9 ± 1.6 | 0.67 | 0.83 |
| Time below 90% oxygen saturation (%) | 8.0 ± 18.0 | 6.0 ± 10.1 | 0.56 | 0.83 |
| Cartwright Index | 1.7 ± 1.1 | 2.2 ± 1.8 | 0.08 | 0.50 |
| Supine time (% of total sleep time) | 36.9 ± 26.8 | 37.2 ± 23.5 | 0.96 | 0.83 |
| Apnea-hypopnea index supine (events/hour) | 22.8 ± 21.0 | 29.3 ± 26.0 | 0.14 | 0.83 |
| Oxygen desaturation index supine (events/hour) | 20.3 ± 19.0 | 27.1 ± 22.9 | 0.14 | 0.83 |
| Heart rate (beats/minute) | 64.4 ± 7.7 | 63.2 ± 7.4 | 0.45 | 0.26 |

**Online Resource Table S3 Univariate Analysis for Predictors of Epworth Sleepiness Scale Responders.** Logistic regression models were applied, and p values were adjusted for multiple comparisons using the Benjamini-Hochberg method.

| Variable | Odds ratio with 95% CI | p value | p value adjusted |
| --- | --- | --- | --- |
| Age (years) | 1.00 (0.97 - 1.03) | 0.91 | 0.99 |
| Gender (female) | 1.71 (0.53 - 6.59) | 0.39 | 0.81 |
| Neck circumference (cm) | 1.01 (0.91 - 1.13) | 0.79 | 0.99 |
| Height (cm) | 1.00 (0.95 - 1.05) | 0.95 | 0.99 |
| Weight (kg) | 1.00 (0.97 - 1.03) | 0.99 | 0.99 |
| BMI (kg/m2) | 1.01 (0.92 - 1.10) | 0.90 | 0.99 |
| Epworth Sleepiness Scale preoperative | 1.15 (1.06 - 1.25) | <0.01 | 0.05 |
| Snoring (VAS) preoperative | 1.08 (0.90 - 1.30) | 0.40 | 0.81 |
| Impaired nasal breathing | 1.83 (0.68 - 5.05) | 0.23 | 0.69 |
| Tonsil grade | 1.35 (0.86 - 2.14) | 0.20 | 0.62 |
| Friedman tongue position | 0.96 (0.65 - 1.41) | 0.84 | 0.99 |
| Friedman stage | 0.50 (0.25 - 0.98) | 0.05 | 0.45 |
| Pharyngeal webbing | 0.90 (0.57 - 1.42) | 0.65 | 0.93 |
| Uvula grade | 1.21 (0.51 - 2.90) | 0.66 | 0.93 |
| Tongue base hyperplasia | 2.40 (0.75 - 9.47) | 0.17 | 0.61 |
| Epiglottis form | 3.14 (0.81 - 15.55) | 0.12 | 0.53 |
| Septal deviation | 0.74 (0.29 - 1.87) | 0.52 | 0.88 |
| Turbinate hypertrophy | 0.94 (0.33 - 2.62) | 0.90 | 0.99 |
| *Preoperative Sleep Testing* |  |  |  |
| Recording time (hours) | 0.75 (0.54 - 1.00) | 0.07 | 0.45 |
| Apnea-hypopnea index (events/hour) | 1.01 (0.99 - 1.03) | 0.35 | 0.81 |
| Apnea index (events/hour) | 1.04 (1.00 - 1.09) | 0.12 | 0.53 |
| **Central Apnea-hypopnea index (events/hour)** | 0.63 (0.38 - 0.90) | 0.04 | 0.45 |
| Oxygen desaturation index (events/hour) | 0.99 (0.97 - 1.01) | 0.43 | 0.84 |
| Mean oxygen saturation (%) | 1.08 (0.86 - 1.38) | 0.50 | 0.88 |
| Time below 90% oxygen saturation (%) | 0.98 (0.95 - 1.02) | 0.34 | 0.81 |
| Cartwright Index | 1.02 (0.83 - 1.34) | 0.85 | 0.99 |
| Supine time (% of total sleep time) | 1.00 (0.99 - 1.01) | 0.85 | 0.99 |
| Apnea-hypopnea index supine (events/hour) | 1.01 (0.99 - 1.03) | 0.26 | 0.73 |
| Oxygen desaturation index supine (events/hour) | 1.00 (0.98 - 1.01) | 0.66 | 0.93 |
| Heart rate (beats/minute) | 1.00 (0.94 - 1.06) | 0.95 | 0.99 |

**Online Resource Table S4 Univariate Analysis for Predictors of Snoring Responders.** Logistic regression models were applied, and p values were adjusted for multiple comparisons using the Benjamini-Hochberg method.

| Variable | Odds ratio with 95% CI | p value | p value adjusted |
| --- | --- | --- | --- |
| Age (years) | 1.00 (0.97 - 1.04) | 0.82 | 0.99 |
| Gender (female) | 1.62 (0.40 - 8.00) | 0.51 | 0.99 |
| Neck circumference (cm) | 0.98 (0.85 - 1.13) | 0.77 | 0.99 |
| Height (cm) | 1.02 (0.97 - 1.08) | 0.35 | 0.99 |
| Weight (kg) | 0.99 (0.96 - 1.02) | 0.37 | 0.99 |
| BMI (kg/m2) | 0.92 (0.83 - 1.02) | 0.13 | 0.99 |
| Epworth Sleepiness Scale preoperative | 0.97 (0.89 - 1.05) | 0.43 | 0.99 |
| Snoring (VAS) preoperative | 0.99 (0.82 - 1.19) | 0.95 | 0.99 |
| Impaired nasal breathing | 1.09 (0.35 - 3.39) | 0.88 | 0.99 |
| Tonsil grade | 0.76 (0.32 - 1.80) | 0.53 | 0.99 |
| Friedman tongue position | 1.77 (0.42 - 7.52) | 0.43 | 0.99 |
| Friedman stage | 0.44 (0.19 - 0.92) | 0.04 | 0.99 |
| Pharyngeal webbing | 1.72 (0.73 - 4.12) | 0.22 | 0.99 |
| Uvula grade | 1.39 (0.52 - 3.73) | 0.51 | 0.99 |
| Tongue base hyperplasia | 1.08 (0.25 - 4.73) | 0.92 | 0.99 |
| Epiglottis form | 0.94 (0.16 - 5.73) | 0.95 | 0.99 |
| Septal deviation | 0.65 (0.21 - 1.98) | 0.45 | 0.99 |
| Turbinate hypertrophy | 1.09 (0.33 - 3.66) | 0.89 | 0.99 |
| *Preoperative Sleep Testing* |  |  |  |
| Recording time (hours) | 1.19 (0.87 - 1.67) | 0.28 | 0.99 |
| Apnea-hypopnea index (events/hour) | 1.01 (0.99 - 1.03) | 0.51 | 0.99 |
| Apnea index (events/hour) | 1.01 (0.98 - 1.05) | 0.63 | 0.99 |
| Central Apnea-hypopnea index (events/hour) | 0.84 (0.55 - 1.05) | 0.27 | 0.99 |
| Oxygen desaturation index (events/hour) | 1.01 (0.98 - 1.04) | 0.51 | 0.99 |
| Mean oxygen saturation (%) | 0.98 (0.74 - 1.28) | 0.87 | 0.99 |
| Time below 90% oxygen saturation (%) | 0.99 (0.95 - 1.03) | 0.63 | 0.99 |
| Cartwright Index | 0.93 (0.61 - 1.39) | 0.70 | 0.99 |
| Supine time (% of total sleep time) | 1.01 (1.00 - 1.03) | 0.09 | 0.99 |
| Apnea-hypopnea index supine (events/hour) | 1.00 (0.98 - 1.02) | 0.95 | 0.99 |
| Oxygen desaturation index supine (events/hour) | 1.00 (0.98 - 1.02) | 0.72 | 0.99 |
| Heart rate (beats/minute) | 0.99 (0.93 - 1.05) | 0.71 | 0.99 |

**Online Resource Fig. S1 Relationship between Epworth Sleepiness Scale Reduction and preoperative Epworth Sleepiness Scale scores.**

A linear regression is indicated by a dashed line (β = 0.79; 95% CI: 0.68 – 0.90; *p* < 0.01).

**Online Resource Fig. S2** **Association of Epworth Sleepiness Scale reduction with Gender.**

Mean reductions for both genders are indicated with a diamond symbol (mean ± standard deviation: males 4.4 ± 4.5, females 7.4 ± 5.2, *p* = 0.03, adjusted *p* = 0.30).

**Online Resource Fig. S3 Relationship between Epworth Sleepiness Scale reduction and preoperative Apnea-Hypopnea Index.**

A linear regression is indicated by a dashed line (β = 0.04; 95% CI: 0.00 – 0.08; *p* = 0.01, adjusted *p* = 0.26).

**Online Resource Fig. S4 Relationship between Epworth Sleepiness Scale reduction and Apnea-Hypopnea Index Reduction.**

A linear regression is indicated by a dashed line (β = 0.07; 95% CI: 0.02 - 0.11; *p* = 0.004).

**Online Resource Fig. S5 Relationship between Snoring Intensity Reduction and preoperative Snoring Intensity.**

A linear regression is indicated by a dashed line (β = 0.73; 95% CI: 0.54 - 0.92; *p* < 0.01).

**Online Resource Fig. S6 Relationship between Snoring Intensity Reduction and Body Mass Index.**

A linear regression is indicated by a dashed line (β = -0.14; 95% CI: -0.27 - -0.01; *p* = 0.04, adjusted *p* = 0.56).

**Online Resource Fig. S7 Association of Snoring Intensity Reduction and Friedman Stage.**

Mean reductions for both genders are indicated with a diamond symbol (mean ± standard deviation: stage 1; 6.7 ± 1.5, stage 2; 5.1 ± 2.3, stage 3; 3.7 ± 3.2, *p* < 0.01, adjusted *p* = 0.09).

**Online Resource Fig. S8 Relationship between Snoring Intensity Reduction and Apnea-Hypopnea Index Reduction.**

A linear regression is indicated by a dashed line (β = 0.007; 95% CI: -0.021 - 0.036; *p* = 0.63).
